# Bringing them back: using latent class analysis to re-engage college stop-outs

**DOI:** 10.3389/fpsyg.2024.1297464

**Published:** 2024-01-29

**Authors:** Cassandra Lynn West, Qi Chen, Nduka Boika

**Affiliations:** ^1^Data, Analytics, and Institutional Research, University of North Texas, Denton, TX, United States; ^2^Department of Educational Psychology, University of North Texas, Denton, TX, United States

**Keywords:** degree completion, stop-out, latent class analysis, person-centered, intervention

## Abstract

Half of the students who begin college do not complete a degree or certificate. The odds of completing a degree are decreased if a student has a low socio-economic status (SES), is the first in a family to attend college (first-generation), attends multiple institutions, stops out multiple times, reduces credit loads over time, performs poorly in major-specific coursework, has competing family obligations, and experiences financial difficulties. Stopping out of college does not always indicate that a student is no longer interested in pursuing an education; it can be an indication of a barrier, or several barriers faced. Institutions can benefit themselves and students by utilizing person-centered statistical methods to re-engage students they have lost, particularly those near the end of their degree plan. Using demographic, academic, and financial variables, this study applied latent class analysis (LCA) to explore subgroups of seniors who have stopped out of a public four-year Tier One Research intuition before graduating with a four-year degree. The findings indicated a six-class model was the best fitting model. Similar to previous research, academic and financial variables were key determinants of the latent classes. This paper demonstrates how the results of an LCA can assist institutions in the decisions around intervention strategies and resource allocations.

## 1 Introduction

Between the years 1993 and 2018, 36 million students enrolled in college but failed to earn a degree or certificate, representing one in six American adults (Shapiro et al., 2014; Steele and Erisman, 2016; Snyder et al., 2019). These figures indicated that half of the students who begin college do not complete a degree or certificate (Mabel, 2017; Shapiro et al., 2018; Snyder et al., 2019). Rates of completion are further decreased if a student has a low socio-economic status (SES), is the first in a family to attend college (first-generation), attends multiple institutions, stops out multiple times, reduces credit loads over time, performs poorly in major-specific coursework, has competing family obligations, and experiences financial difficulties (Shapiro et al., 2014; Mabel et al., 2017; Forrest Cataldi et al., 2018). While a third of these students stop out after only one semester, a significant portion of students stop out after having completed at least three-quarters of the credits necessary for their degree (Shapiro et al., 2014; Mabel and Britton, 2017).

Having a post-secondary education credential is becoming increasingly important for both the individual and society. Earning a degree positively affects an individual in many ways, such as increased employment opportunities, job security, higher wages, job satisfaction, health and pension benefits, and social mobility (Snyder and Dillow, 2011; Lane et al., 2012; Baum et al., 2013; Hagelskamp et al., 2013; Shapiro et al., 2014; Steele and Erisman, 2016; Mitchell et al., 2017; Snyder et al., 2019). A degree does not guarantee economic success, but it greatly increases the odds; when it comes to salary and wages over a lifetime, having a college degree makes a substantial difference (Gladieux and Perna, 2005; Baum et al., 2013; Shapiro et al., 2014; Snyder et al., 2019). Even the completion of some college coursework leads to 13% higher wages for individuals when compared to high school graduates, and that gap in wages continues and grows with time spent in the workforce (Baum et al., 2013; U.S. Department of Education, 2015; Snyder et al., 2019). Additionally, individuals with a degree are more likely to have employment stability and less likely to suffer from unemployment, even in dire economic situations, such as the 2008 recession (Lane et al., 2012; Snyder et al., 2019).

A college degree not only protects against unemployment, but also is increasingly necessary for the jobs of the future. Having an educated population provides the country with economic development, increased global competitiveness, and a stronger workforce (Lane et al., 2012; Hagelskamp et al., 2013; Steele and Erisman, 2016; Mitchell et al., 2017). Technological advances and rapid innovations created a job market that demands higher education credentials, and the country faces a major shortfall (Lane et al., 2012; Mitchell et al., 2017; Texas Higher Education Foundation, 2017). It is predicted that approximately 60% or more of the new jobs created by 2030 will require some level of higher education (Texas Higher Education Foundation, 2017), but currently, less than 40% of the adult population in the US holds an associate’s degree or higher (Gladieux and Perna, 2005; Shapiro et al., 2014).

As a result of significant changes to the financial landscape of higher education, out-of-pocket costs are one of the most pressing concerns for students, influencing their enrollment decisions and completion progress, and a heavy reliance on sources of financial aid (Soares et al., 2016; Mitchell et al., 2017; Walizer, 2018). In the last decade, state funding per full-time equivalent students (FTE) at public institutions declined by 25%, while the cost of tuition, fees, room, and board rose by 31% (Flores and Shepherd, 2014; Deming and Figlio, 2016; Soares et al., 2016; McFarland et al., 2019; Snyder et al., 2019). Students are burdened by ever-increasing education costs while state and federal aid decreases, and relying on loans to pay for an education is a risky long-term financial decision, especially for non-completers (Mitchell et al., 2017; Itzkowitz, 2018; Safier, 2018). Additionally, students are increasingly mobile with their studies, attending multiple institutions while completing a degree. Although convenient for students, this mobility comes at a cost. Less than a third of students transferring between institutions can use all of their earned credits, one-third loses an average of 13 (credits equivalent to one full semester of coursework), and the remaining third loses everything (Peter and Forrest Cataldi, 2005; Hossler et al., 2012; Simone, 2014; US GAO, 2017; Giani, 2019). The loss of time and finances spent to earn these credits are substantial impediments to completion. Increased time to obtain a degree, attending multiple institutions, and graduating with loan debt come at great costs to students and jeopardize long-term economic gains (Goldrick-Rab et al., 2014; Shapiro et al., 2016; Mitchell et al., 2017).

At the same time, institutions face pressure from external entities: not only are they judged and compared for retention and completion metrics, but also funding to institutions is increasingly tied to these performance metrics (Tandberg and Hillman, 2014; Deming and Figlio, 2016). The rise in performance-based funding requires institutions to rethink structures, resources, and programs and shift their focus from enrollment to long-term student success (Soares et al., 2016; Lumina Foundation, n.d.). Time to degree is one of the most prominent outcomes for which an institution is measured, despite criticism for its limited scope of inclusion (Shapiro et al., 2014). For four-year institutions, time to degree is measured for the traditional first time in college (FTIC) freshman who is attending full-time and earns a degree within four or six years from the same institution (Shapiro et al., 2018; Snyder et al., 2019). That archetype of students is becoming more of the minority rather than the rule, with 43% of bachelor’s degree earners enrolling in more than one institution along the way (Peter and Forrest Cataldi, 2005; Shapiro et al., 2016). These measurements fail to account for the varied pathways students take to completion.

In addition, changing demographics in the years to come will pose challenges for reaching the educational goals of the country (Gladieux and Perna, 2005; Bransberger, 2017; Grawe, 2019). Nationally, the number of students graduating from high school will decrease significantly due to widespread declining birth rates in the United States; the 2018 birth rate was the lowest in history at 59.1 births per 1,000 females aged 15–44 (Martin et al., 2019). Furthermore, the coming generations of college students are going to be more ethnically diverse and come from populations that have traditionally been first-generation, economically disadvantaged, and academically underserved (Gladieux and Perna, 2005; Bransberger and Michelau, 2016; Bransberger, 2017; Grawe, 2019). Finding ways to better serve these students and close the enrollment and completion gaps will be crucial for institutions to meet performance metrics goals. This is also critical for producing an educated population and ensuring the United States has a strong workforce trained for the jobs of the future.

In order to meet the challenges ahead, it will be important for institutions to understand the specific completion barriers faced by students as they seek to enroll and graduate students within the rapidly changing education landscape. Institutions need to focus on stopping student attrition or intervening after it has occurred. Stopping out of college does not always indicate that a student is no longer interested in pursuing an education; it can be an indication of a barrier or several barriers faced. Institutions can benefit themselves and students by re-engaging students they have lost, particularly those near the end of their degree plan.

Students stop out of college for a variety of reasons. Some of the most significant reasons students cite center around caregiving responsibilities, financial difficulties, and academic difficulties (Donhardt, 2012; Lane et al., 2012; Hagelskamp et al., 2013; Schulte, 2015; Steele and Erisman, 2016; Mabel and Britton, 2017). Increasing work and family obligations directly conflict with the time needed to pursue an education and are the most frequently cited challenges for adult students. However, many institutions do not have data about students’ family or work situations or the capacity to mitigate these issues. Often, institutions do not even have the opportunity to work with a student on the barriers they face; the student simply stops enrolling without accessing various campus resources. To formulate interventions to reengage a student who has stopped out, institutions need to focus on the available data and address the issues within their control or scope of impact. For instance, knowing a student is a first-generation or a transfer student is useful because there are resources built around these specific student characteristics. Further, institutions can investigate various academic and financial variables, and look out for combinations of these variables that form barriers for degree completion.

One set of variables available to an institution and within their realm of influence relates to a student’s academic record. The student’s academic performance, as well as their academic pathway, form academic barriers to reentry. Depending on the length of the enrollment gap, a student who stopped out may need to reapply to the institution to reenroll. Adult students, especially those who are also first-generation, cite a lack of information about re-entering post-secondary education (Donhardt, 2012; Lane et al., 2012; Hagelskamp et al., 2013). Obtaining that information is more difficult if institutions only operate within typical business hours; adult learners with substantial work and family obligations cannot access the assistance needed to navigate the re-application process (Hagelskamp et al., 2013). Aside from the act of re-enrolling, students may have other barriers within their previous enrollments that prevent progress. Difficulty in completing upper-division courses, accumulation of excess semester credit hours, and the loss of credits with transferring are common academic barriers students face to completion and reentry (Donhardt, 2012; Lane et al., 2012; Hagelskamp et al., 2013; Fematt et al., 2019). Additionally, students with work and family obligations need flexible learning environments to balance the competing demands; some of the needed coursework may not be in an online or other accessible format (Lane et al., 2012; Hagelskamp et al., 2013; Fematt et al., 2019). Intuitions can gain insight into the academic situation of a student who has stopped out by examining their total hours accumulated, changes in performance, signs of academic decline, if the student was last enrolled in coursework that matches their classification (e.g., Juniors or Seniors in upper-division coursework), and the student’s academic standing during last enrollment.

The second set of variables an institution can focus on revolves around student finances. The cost of education and the stress of paying weigh heavily on students’ attempts to reenter college. Many students who have stopped out no longer qualify for financial aid or scholarships and are worried about taking on a loan debt (Donhardt, 2012; Hagelskamp et al., 2013; Mabel, 2017; Mabel and Britton, 2017). One financial factor that is an immediate barrier to re-enrollment is debt owed to the institution; students who owe even a small amount from a previous term are unable to register for classes, and these debts often end up with debt collectors, which increases the students’ financial burden (Clark, 2009; Douglas-Gabriel, 2016; Scobey, 2017). Institutions can thus examine the student’s financial situation during their last enrollment to gain some insights into the specific financial barriers at play. Institutions have access to financial information through the Free Application for Federal Student Aid (FAFSA) and student records. It will help to form a picture of the students’ financial condition by examining if the student owes the institution money, were Pell Grant eligible in prior terms, left the institution meeting satisfactory academic progress for financial aid, and borrowed loans for previous work at the institution. Uncovering and understanding the financial obstacles faced by students are crucial for reengaging them.

Institutions need to understand how all the academic and financial variables are operating together to work against a student’s degree completion. A student rarely has a singular barrier to degree completion; more often, it is a combination of factors (Donhardt, 2012; Hagelskamp et al., 2013; Steele and Erisman, 2016; Mabel and Britton, 2017). How these combinations of factors present themselves are unknown to institutions. Data mining and employing innovative data analysis techniques are critical first steps in helping institutions understand and serve these students more effectively (Lane et al., 2012; Soares et al., 2016).

As institutions seek to make data-informed decisions to meet the challenges ahead, they must consider new ways to approach old problems. Variable-centered approaches look for overarching trends within a population and currently dominate the landscape of analyses in educational, psychological, and other social sciences research (Coladarci and Cobb, 2013; Howard and Hoffman, 2017). These types of analyses seek to answer questions along the lines of what factors place a student most at risk for dropping out. Although useful, variable-centered analyses assume the associations among variables are consistent across the population (Collins and Lanza, 2010; DiStefano, 2012; Marcoulides and Heck, 2013; Denson and Ing, 2014; Weerts et al., 2014; Malcom-Piqueux, 2015; Gray, 2019). Some researchers in the fields of education, behavioral science, and social sciences believe that this assumption of homogeneity within a population has led to conflicting results or inconsistencies among studies with cross-sectional and longitudinal data (Marcoulides and Heck, 2013). Variable-centered methods are useful when exploring inter-individual differences (e.g., differences in SAT scores between groups of students) but not intra-individual subtleties (e.g., how behavioral profiles of an individual change over time) (DiStefano and Mindrila, 2013). This approach can be helpful for prevention efforts, such as knowing the most common variables associated with attrition. However, they are not useful in understanding how the unique barriers experienced by a student are working together to prevent progress.

In contrast, person-centered approaches are considered holistic because they assume that there are subgroups/subpopulations of individuals with similar response patterns and the variables’ impact the on the outcome is heterogeneous across the different populations (Collins and Lanza, 2010; DiStefano, 2012; Marcoulides and Heck, 2013; Denson and Ing, 2014; Malcom-Piqueux, 2015; Gray, 2019). Person-centered analyses are particularly useful when the size and structure of the subgroups/subpopulations are unknown (Lanza et al., 2013). Person-centered methods are widely used in health and social sciences intervention work. Researchers use these methods to identify clusters of symptoms to treat or partition larger groups into different behavioral profiles (Collins and Lanza, 2010; Lanza and Rhoades, 2013; Butera et al., 2014). The ability to examine the comorbidity of multiple risk factors within the subpopulations is more meaningful than attempting to treat each risk factor as if it is operating alone (Lanza and Rhoades, 2013). Unfortunately, these approaches are underutilized in higher education and are nearly absent from intervention work within the field. The application of person-centered approaches can help uncover particular combinations or intersections of variables that are characteristic of hidden subgroups within a larger heterogeneous population. Person-centered approaches can reveal patterns that go unnoticed when using variable-centered approaches. Once a larger population of students is partitioned into smaller and characteristically different subgroups, institutions can tailor interventions to the specific needs of each subgroup, rather than applying broad interventions to the entire population. For example, an institution would likely be more successful in re-enrolling students if separate intervention strategies were built around the unique combination of factors that lead to the student leaving rather than a broad re-enrollment campaign that ignores the many barriers that students face.

Several person-centered analyses aim to classify individuals into previously unknown groups based on shared characteristics, most of which fall under the umbrella of cluster analysis. Cluster analysis is a technique that subsumes various methods for partitioning a larger group of subjects into subgroups and consists of hierarchical and partitional methods (Borden, 2005; Chen, 2008; Chen et al., 2010; DiStefano and Mindrila, 2013). Traditional clustering techniques lack statistical and objective methods for determining the appropriate number of clusters; relying on heuristics, the researcher must determine the appropriate number of clusters for a solution (Borden, 2005; Pastor et al., 2007; DiStefano, 2012; DiStefano and Mindrila, 2013). One criticism of traditional cluster analysis is that the technique is data-driven and heavily dependent on variable selections or initial splits in the data (Borden, 2005; Pastor et al., 2007; DiStefano, 2012; DiStefano and Mindrila, 2013). Additionally, these techniques do not allow for a case or individual to belong to more than one group, and the solutions are sample dependent without the ability to infer to populations (Borden, 2005; Pastor et al., 2007; DiStefano, 2012; DiStefano and Mindrila, 2013). These issues are remedied by the use of finite mixture models.

Finite mixture modeling (FMM) is a partitional clustering technique. FMM assumes the existence of latent subgroups/classes within a population and the model parameters differ among those unobserved latent classes (Chen, 2008; Marcoulides and Heck, 2013). FMM analyses include techniques such as latent class analysis, latent profile analysis, latent transition analysis, growth mixture modeling, mixture factor analysis, and mixture structural equation modeling (Chen, 2008; DiStefano, 2012; Marcoulides and Heck, 2013). These methods differ from traditional cluster analysis in that they are model-based; seeking to determine a model that maximizes homogeneity within subgroups/classes and maximizes heterogeneity between classes. Additionally, in FMM the goal is to optimize the fit of the model to the data, which allows for flexibility of group membership (DiStefano, 2012; Lanza et al., 2013; Marcoulides and Heck, 2013). Further, FMM can be applied to different kinds of data, including continuous or categorical and cross-sectional or longitudinal (Chen, 2008; Collins and Lanza, 2010; Marcoulides and Heck, 2013).

This paper used one particular method within the FMM framework, latent class analysis (LCA). LCA is a subgrouping technique used heavily in the health and behavioral fields that lends itself well to the exploration of subgroups within higher education (Collins and Lanza, 2010; Boscardin, 2012; Denson and Ing, 2014; Malcom-Piqueux, 2015; Fematt et al., 2019; Gray, 2019). LCA is appropriate for cross-sectional samples with categorical variables. The primary goal is to arrive at an array of subgroups, referred to as latent classes, which represent different observed response patterns (Collins and Lanza, 2010). Since LCA falls under the umbrella of FMM, the prevalence for each class and the error associated with each variable measuring the latent classes can be estimated. Within the analysis, researchers can identify items that indicate the classes well and estimate the probabilities of item endorsement within the latent classes (conditional item response probabilities). Additionally, a researcher can identify covariates and grouping variables that help explain class membership and classify individuals correctly into each latent class (Collins and Lanza, 2010; Malcom-Piqueux, 2015).

In fields that use LCA, researchers and practitioners apply the results of the analysis to individualize interventions and increase intervention effectiveness (Boscardin, 2012; Rosato and Baer, 2012; Lanza et al., 2013; Lanza and Rhoades, 2013; Malcom-Piqueux, 2015). The ability to create subgroups based on the intersections of multiple observed variables is critical for intervention work, matching the individual to the most appropriate treatment (Boscardin, 2012; Rosato and Baer, 2012; Lanza and Rhoades, 2013). Higher education often works with limited resources and staff to carry out interventions. Finding ways to maximize the effectiveness of programs will become increasingly important with the challenges ahead (Lane et al., 2012; Soares et al., 2016). As institutions look to engage students and approach old problems in new and meaningful ways, LCA can facilitate the necessary shift in perspective and tactics.

There are relatively fewer published papers using latent class analysis (LCA) in higher education research, but those that do exist underscore the utility of this method. Each application of LCA in higher education added information and perspectives that were not attainable through variable-centered approaches. Boscardin (2012) used LCA to redefine the process for identifying medical students for remediation. Using LCA, Boscardin (2012) found three groups of students with qualitatively different performance profiles on the Clinical Performance Examination, two of which were identified for remediation based on deficiencies in separate areas. This technique identified an additional 24% of students for remediation. After the identification of the specific deficits, targeted interventions occurred without the use of additional resources. Malcom-Piqueux (2015) sought to explore inequities in college financing strategies. Using LCA, she uncovered three unique payment patterns among post-secondary students: self-support, parental-support, and distributed-support, and examined how these strategies were shaped by race and ethnicity. Gray (2019) examined the human and social capital of adult immigrants and uncovered a gross mismatch between the educational needs and the available educational resources. Denson and Ing (2014) and Fematt et al. (2019) used LCA to identify subgroups of students and advocate for targeted interventions. Denson and Ing (2014) offered a pedagogical application of LCA within higher education regarding students’ pluralistic orientation. Fematt et al. (2019) examined whether meaningful subgroups of transfer students emerged based on their response patterns to measures of academic and social adjustment. These research studies partitioned larger groups of students into meaningful subgroups, allowing administrators and institutions to tailor their courses, curricula, and services to specific issues uncovered by LCA.

The purpose of the current study was to expand the use of latent class analysis in higher education and demonstrate the utility of the technique in intervention planning. Specifically, the study used LCA to explore subgroups of students who have stopped out of college before graduating with a four-year degree. Depending on the size of the institution, the number of stopped-out students can be quite large. The researchers employed LCA to uncover several smaller subgroups of students from a larger sample, with the goal of helping the institution focus resources on the students most likely to return and guide the intervention strategies used to re-enroll students. Different combinations of barriers to re-enrollment require different actions or intervention strategies, and some of the combinations are easier to address. Segmenting the sample of stop-outs into meaningful groups with varying needs allows institutions to maximize resources by identifying which students to focus attention, and then matching students to specific resources and paths to re-enrollment. The research questions included: (1) Are there distinct subgroups/classes of students who have stopped out based on their response patterns to academic, financial, and demographic variables? (2) What are the prevalence and characteristics of each class/subgroup within the sample?

## 2 Methods and materials

### 2.1 Location and participants

The study occurred at a large four-year public Tier One Research institution in Texas. The institution’s overall four-year graduation rate is 41.5% and the six-year rate is 61.2%; both rates were close to the state graduation rates of 40.1% for four-year and 63.6% for six-year for public institutions (Texas Higher Education Accountability System, 2020). The institution seeks to increase its graduation rate metrics to improve student success and help Texas reach goals associated with 60x30TX, a plan aimed at increasing statewide post-secondary credentials (Stedman et al., 2019). One way to accomplish this goal is to re-enroll students who left the institution with a substantial amount of coursework completed.

IRB approval was obtained from the university to perform secondary analysis on the academic records of former students. The sample consisted of students enrolled at the institution between Fall 2015 and Fall 2019 who reached the classification of senior but failed to graduate or re-enroll. A total of 3,662 students met these criteria, representing 8.70% of all seniors enrolled at the institution during the period of interest. For variables obtained in a continuous format, univariate (z-scores) and multivariate (Mahalanobis distance) were performed. Nine participants were removed for extreme values and on age and number of semester credit hours earned (*z* > 5.0), bringing the final data set to 3,653. The stopped-out seniors were significantly older (*M* = 26.9 years, *SD* = 7.02; *t*(45,719) = 21.53, *p* < 0.0001) than the overall senior population (*M* = 24.71 years, *SD* = 5.85). Additionally, there were statistically significant differences in gender and race/ethnicity for stopped-out seniors when compared to the overall senior population (see Table 1); the stopped-out seniors were more heavily male and over-represented by students identifying as Black or two or more races.

**Table 1:** Frequencies and chi-squared results for ethnicity and gender.

Demographic variable	Stopped-out seniors		All seniors		ꭓ^2^(1)
	*n*	%	*n*	%	
Ethnicity					
Asian	189	5.17%	2,303	5.47%	0.59
Black	658	18.01%	5,451	12.96%	76.66**
Hispanic	823	22.53%	9,991	23.75%	2.77
Two or more races	183	5.01%	1,696	4.03%	8.19*
White	1,622	44.40%	20,626	49.03%	28.84**
Other	178	4.87%	2,001	4.76%	0.09
Gender					
Male	2,026	55.46%	19,580	46.54%	107.30**
Female	1,627	44.54%	22,488	53.46%	107.30**

*p < 0.05, **p < 0.0001.

### 2.2 Description of variables

Three sets of variables were obtained for this study, demographic, academic, and financial. The Office of Institutional Research compiled all data items through institutional records. The records contain both self-reported data (race/ethnicity, gender, and first-generation status) and items that are contained in official institutional records (all remaining items). With the exception of gender and race/ethnicity, all variables were included in the LCA. Demographic measures included gender (male/female), race/ethnicity (Asian, Black, Hispanic, White, Two or more, and Other), non-traditional age (dummy variable with 1 indicating a student was 25 years or older, and 0 otherwise), and first-generation status (1 indicating that neither of the student’s parents had college experience, 0 the parents who had some college experience, and 2 as unknown).

Academic variables reflected the student’s overall record and status during their last semester of enrollment. Variables related to course taking behavior included full-time enrollment status during last semester (dummy variable with 1 indicating full-time, and 0 otherwise), senior level coursework (dummy variable with 1 indicating if the student was enrolled in at least one 4,000 level course during their last semester, and 0 otherwise), and academic decline (dummy variable with 1 indicating a student earned one or more grades of D of F during their last enrollment, and 0 otherwise). Additional academic variables included excess hours (dummy variable with 1 indicating if a student accumulated more than 180 semester credit hours, and 0 otherwise), good academic standing (dummy variable with 1 indicating good, and 0 poor) indicate, and student type upon entering the university (1 indicating a student who enrolled as a freshman/native student, and 0 for transfer students).

Financial variables were included to assess debt status and aid used during prior enrollments. This included institutional debt (dummy variable with 1 indicating the student had a balance on their account, and 0 otherwise), Pell eligible (dummy variable with 1 indicating the student had been Pell eligible at any time between 2011 and 2019, and 0 otherwise), and loan debt (dummy variable with 1 indicating the student borrowed money during their time at the institution, and 0 otherwise).

Only two variables had missing data within institutional records, first-generation status and Pell eligibility. First-generation status was calculated from non-compulsory items on the university application, some students chose not to report parental education, and as a result were coded as unknown. Pell eligibility was determined by information on a completed FAFSA; some students elected not to complete a FAFSA, and therefore did not have data around Pell eligibility. Students without any FAFSA/Pell eligibility information between 2011 and 2019 were coded as unknown. After coding “unknown” for Pell eligibility and first-generation status and including “unknown” in the analysis, the data set contained no missing data.

### 2.3 Limitations

Limitations to this study related to the variables included in the analysis have implications for future research. Students’ academic and personal lives are complex and the data available to institutions is often limited, but this study demonstrated how to work within these limitations. Access to demographic, academic, and financial data is readily available to institutional researchers. Although additional data related to concepts such as personality, cognitive ability, and motivation, would be ideal in understanding all the mechanisms that influence a student’s higher education journey, they simply are not available on a large scale at most institutions. Nor are data readily available related to key aspects of life that are directly compete with a student’s attention to education such as work demands, family obligations, or health status. What is available will differ greatly between institutions. Attempting to collect all relevant data can lead to analysis paralysis and missed opportunities to work with what *is* available. The reality is that we will never have access to all the factors influencing a student’s ability to continue their education. However, a goal of this study was to navigate within these limitations and maximize the usefulness of what is available.

### 2.4 Analytical approach

Latent class analysis is a person-centered approach used to explore the underlying heterogeneity of a population, sample, or set of data. This allows for a deeper understanding and a more accurate description of the relationships that exist in the data by relaxing the assumption that the relationships are the same for all individuals within the larger group (Nylund-Gibson et al., 2014). LCA is a statistical approach that can be used to discover latent categories of students that are too complex to observe or explore through variable-centered approaches. LCA differs from other subgrouping techniques, such as traditional cluster analysis, in that it is model-based; seeking to determine a model that maximizes homogeneity within subgroups/classes and maximizes heterogeneity between classes. Additionally, the goal is to optimize the fit of the model to the data, which allows for flexibility of group membership (DiStefano, 2012; Lanza et al., 2013; Marcoulides and Heck, 2013).

Maximum Likelihood Estimation (MLE) is the go-to method for estimating parameters in Latent Class Analysis (LCA) due to its reliability and efficiency. It is widely used in social, behavioral, and health sciences, providing consistent, unbiased, and asymptotically efficient estimates (Lehmann and Casella, 1998; Muthén and Muthén, 2002; Serfling, 2009). MLE achieves the Cramér-Rao lower bound, minimizing asymptotic variance and demonstrating high relative efficiency (Lehmann, 1949). These properties make MLE a robust and effective choice for latent class modeling, as confirmed in our analyses conducted with Mplus 8 (Muthén and Muthén 1998, 2017).

A combination of past research, fit statistics, and model checking measures are used to determine the number of latent classes. Fit indices help to approximate the correct number of classes and model-checking measures examine the overall classification of individuals within classes. Fit indices fall into four categories; information-based criterion (IC), nested model likelihood ratio tests, goodness of fit measures, and classification-based statistics (Chen et al., 2017). The most commonly used and recommended for LCA include Akaike’s information criterion (AIC), Bayesian information criterion (BIC), and sample size adjusted BIC (SABIC) for IC based fit statistics, and the Vuong-Lo–Mendell–Rubin likelihood ratio test (VLMR) and the bootstrap likelihood ratio test (BLRT) for the nested model likelihood ratio tests (Nylund-Gibson et al., 2014; Nylund-Gibson and Choi, 2018). Conflicting information among fit indices is common. When this occurs, the interpretability and substantive meaningfulness of the classes guide decisions around selection of the best fitting model (Muthén, 2003). In addition to fit statistics, entropy and average posterior probabilities (AvePP) help determine how well the model classifies individuals into their respective latent classes. Lastly, class homogeneity and class separation are examined through conditional item response probabilities (the likelihood of endorsing each item as a function of class membership), to assess the overall classification certainty. Class homogeneity and separation indicate how similar individuals within classes are to each other and how distinguishable the classes are from each other, respectively. LCA can be performed in a multitude of software programs, including SAS, Mplus, LatentGold, and R. Mplus 8.4 was used for this study (Muthén and Muthén, 2017).

Although the stop-out population has not been examined with this particular analysis approach, past research has shown that students who stopped out have either academic barriers, financial barriers, a combination of the two, or no clear barriers based on academic or financial data. How these barriers are uniquely combined for this population is unknown, but it was hypothesized that a 4-class solution will fit the data resulting in the following classes: (1) students with primarily academic barriers, (2) students with primarily financial barriers, (3) students with a combination of academic and financial barriers, and (4) a class of students with no significant barriers academic or financial barriers (Donhardt, 2012; Lane et al., 2012; Hagelskamp et al., 2013; Schulte, 2015; Steele and Erisman, 2016; Mabel and Britton, 2017). A combination of past research findings, interpretability and meaning of the latent classes, fit statistics, and model checking measures were used to determine the number of latent classes.

## 3 Results

Data analysis occurred in two steps. First, descriptive statistics were conducted on the academic, financial, and remaining demographic variables. Table 2 demonstrates the proportion of endorsement for each category of the variables included in the analysis. Next, a series of latent class analyses were conducted using a model testing strategy that began with a model consisting of one latent class and each subsequent model was increased by one latent class until fit statistics and model checking measures indicated an appropriate solution (Collins and Lanza, 2010; Marcoulides and Heck, 2013; Nylund-Gibson and Choi, 2018).

**Table 2:** Descriptive statistics for model predictors.

Variables	% of participants		
	Yes	No	Unknown
Non-traditional age	49.93	50.07	
First generation	43.91	41.14	14.95
Full-time enrollment	39.94	60.06	
Senior level coursework	55.71	44.29	
Excess credit hours	26.01	73.99	
Good academic standing	68.38	31.62	
Academic decline	62.20	37.80	
Institutional debt	38.43	61.57	
Pell eligible	62.52	14.76	22.71
Borrowed loans	64.91	35.09	
Student type	Native	Transfer	
	27.76	72.24	

### 3.1 Model selection

To address the research question of determining the existence of latent classes within the overall stopped-out seniors group, a set of latent class analyses were performed for one to eight classes. Beginning with a one-class model and increasing the classes by one in each subsequent model allowed for examining whether the absolute and relative fit indices supported the model with additional classes. Whether the addition of another class significantly improved the model is determined by decreasing values for AIC, BIC, and SABIC and significant value of *p*s for the ꭓ^2^ tests of LMR-LRT, VLMR-LRT, and BLRT. Table 3 presents a summary of the fit indices for latent class models with one to eight classes. Although the AIC, BIC and SABIC continued to decrease with each subsequent class and the BLRT *value of p* remained significant, non-significant *p*-values for LMR-LRT and VLMR-LRT indicated support for a six-class solution. Table 3 also demonstrates diminishing decrement in the value of BIC beyond six classes, which is an indication that the six classes solution is best (Petras and Masyn, 2010). In addition, the latent classes extracted beyond six classes became difficult to distinguish from other existing classes or identify unique intervention strategies to employ. Parsimony and substantive interpretation of the latent classes increased support for the six-class solution.

**Table 3:** Summary of latent class analysis fit indices with 1–8 latent classes.

Classes	Loglikelihood	Number of parameters	AIC	BIC	SABIC	LMR-LRT *p* value	VLMR-LRT *p* value	BLRT *p* value	Entropy
1	−28,278.20	13	56,582.39	56,663.03	56,621.73				
2	−27,285.04	27	54,624.09	54,791.58	54,705.78	< 0.001	< 0.001	< 0.001	0.803
3	−26,784.02	41	53,650.05	53,904.38	53,774.10	< 0.001	< 0.001	< 0.001	0.873
4	−26,571.12	55	53,252.24	53,593.43	53,418.66	< 0.001	< 0.001	< 0.001	0.786
5	−26,407.55	69	52,953.10	53,381.13	53,161.88	< 0.001	< 0.001	< 0.001	0.773
6	−26,300.64	83	52,767.28	53,282.16	53,018.42	0.006	0.005	< 0.001	0.751
7	−26,218.68	97	52,631.37	53,233.09	52,924.87	**0.805**	**0.805**	< 0.001	0.747
8	−26,162.67	111	52,547.34	53,235.91	52,883.20	**0.781**	**0.812**	< 0.001	0.721

Bolded values highlight non-significant p-values for LMR-LRT and VLMR-LRT, which indicate support for a six-class solution.

Along with fit indices, Tables 4, 5 present the results for the six-class solution and show a high degree of within-class homogeneity and separation for the six latent classes. Table 4 presents the average posterior probabilities (AvePP) which provide information on how well the model classified individuals into their most likely class. AvePP values of >0.70 indicate a high degree of class separation (Nylund-Gibson and Choi, 2018). Table 5 shows the class labels, prevalence rates, relative size, and the item response probabilities for each variable within the classes. High within-class homogeneity indicates that respondents in a particular class are likely to respond similarly to an item and is a critical determining criterion for latent class analysis. Items with response probabilities >0.70 define or epitomize the class and demonstrate class homogeneity (Masyn, 2013). Together, these evidences support a six-class model of stopped-out seniors.

**Table 4:** Classification probabilities: senior stop-outs six-class solution.

	1	2	3	4	5	6
1: Distracted, self-pay	**0.844**	0.025	0.057	0.026	0.045	0.003
2: Distracted, high financial need	0.028	**0.764**	0.086	0.015	0.000	0.106
3: Academic decline, high financial need	0.018	0.005	**0.929**	0.000	0.006	0.043
4: *Swirlers*	0.112	0.071	0.000	**0.713**	0.055	0.049
5: Academic distress, self-pay	0.085	0.004	0.033	0.072	**0.769**	0.038
6: Academic distress, high financial need	0.001	0.093	0.114	0.006	0.008	**0.778**

Values indicate probabilities of most likely class membership (column) by latent class modal assignment (row). Bolded values along the diagonal indicate average posterior probabilities (AvePP). Values off of the diagonal indicate the average probability of membership in an alternative latent classes given most likely class membership.

**Table 5:** Class prevalence, size, and item-response probabilities.

		Class 1: distracted, self-pay	Class 2: distracted, high financial need	Class 3: academic decline, high financial need	Class 4: *Swirlers*	Class 5: academic distress, self-pay	Class 6: academic distress, high financial need
Prevalences		13.93%	19.66%	32.55%	7.25%	7.97%	18.64%
Estimated size (*n*)		509	718	1,189	265	291	681
Item-response probabilities							
First-generation	Yes	0.314	0.489	0.531	0.260	0.224	0.501
	No	0.464	0.367	0.399	0.399	*0.600*	0.365
	Unknown	0.222	0.143	0.071	0.342	0.175	0.134
Non-traditional age	Yes	0.431	**0.894**	0.000	**1.000**	0.310	**0.744**
	No	0.569	0.106	**1.000**	0.000	**0.690**	0.256
Admit type	Native	0.270	0.145	0.551	0.103	0.214	0.109
	Transfer	**0.730**	**0.855**	0.449	**0.897**	**0.786**	**0.891**
Good academic standing	Yes	**0.992**	**0.874**	**0.776**	**0.657**	0.338	0.256
	No	0.008	0.126	0.224	0.343	**0.662**	**0.744**
Senior level	Yes	0.441	0.537	*0.632*	0.424	0.553	*0.605*
	No	0.559	0.463	0.368	0.576	0.447	0.395
Excess hours	Yes	0.040	0.363	0.069	**0.758**	0.196	0.409
	No	**0.960**	*0.637*	**0.931**	0.242	**0.804**	0.591
Full-time enrollment	Yes	0.242	0.199	0.537	0.148	0.559	0.570
	No	**0.758**	**0.801**	0.463	**0.852**	0.441	0.430
Academic decline	Yes	0.174	0.401	**0.693**	0.503	**0.941**	**1.000**
	No	**0.826**	0.599	0.307	0.497	0.059	0.000
Pell eligible	Yes	0.193	**0.860**	**0.740**	0.224	0.071	**0.907**
	No	0.137	0.140	0.259	0.008	0.053	0.091
	Unknown	**0.669**	0.000	0.002	**0.767**	**0.877**	0.002
Institutional debt	Yes	0.120	0.442	0.393	0.346	0.310	0.549
	No	**0.888**	0.558	*0.607*	**0.654**	**0.690**	0.451
Loans borrowed	Yes	0.065	**0.906**	**0.886**	0.187	0.073	**0.852**
	No	**0.935**	0.094	0.114	**0.813**	**0.927**	0.148

Item-response probabilities indicate the probability of a member of that latent class endorsing an item. For example: 31.4% of individuals in Class 1 indicated Yes for first-generation status. Values > 0.66 and greater are bolded to indicate strong item endorsement, between 0.60 and 0.66 are italicized to show moderate item endorsement.

An examination of Bivariate Residuals (BVR) across 11 key LCA variables was conducted. The objective was to discern any relationships among these variables that extend beyond the latent class structure. The BVR chi-square values exceeding the threshold of 4 are considered indicative of significant associations (Nagelkerke et al., 2017). Our findings revealed 12 out of 55 pairs of indicator variables with BVR exceeding 4, and 5 out of the12 high associations involved variables that were not helpful in determining the latent classes, namely, first generation status and senior level coursework.

### 3.2 Latent class characteristics

The latent classes were characterized by demographic, academic, and financial variables. The classes ranged in sizes from 7.25% (Class 4, *n* = 265) to 32.55% (Class 3, *n* = 1,189) of the total sample (see Table 5). In Table 5, the bolded response probabilities indicate variables that were strongly endorsed by each class which also indicates how similar individuals within the class are to each other. Alternatively, the odds ratios in Table 6 indicate variables that are critical to the degree of separation between the classes (odds ratios >5 or < 0.2) and how dissimilar individuals are across classes. These tables combined reveal the items that did not contribute to formation of the latent classes. Specifically, first-generation status and senior-level courses were the least helpful in determining the latent classes; item endorsement rates for both variables across all six classes failed to reach the 0.7 threshold while also failing to contribute to class separation. In contrast, good academic standing, non-traditional age, freshman/native student, excess hours, enrollment status, academic decline, Pell eligibility, institutional debt, and a history of loan borrowing were endorsed strongly throughout the classes and contributed significantly to the distinction between classes.

**Table 6:** Degree of class separation.

		Class 1 vs. 2	Class 1 vs. 3	Class 1 vs. 4	Class 1 vs. 5	Class 1 vs. 6	Class 2 vs. 3	Class 2 vs. 4	Class 2 vs. 5	Class 2 vs. 6	Class 3 vs. 4	Class 3 vs. 5	Class 3 vs. 6	Class 4 vs. 5	Class 4 vs. 6	Class 5. vs 6
First-generation	Yes	1.307	2.865	2.735	3.226	0.825	2.193	2.093	2.469	0.631	0.550	1.126	0.288	1.180	0.302	0.256
	No	1.821	3.354	3.099	**6.820**	2.441	1.842	1.703	3.745	1.340	0.924	2.033	0.728	2.201	0.788	0.358
Non-traditional		**0.000**	**0.000**	**0.000**	**0.000**	**0.000**	3.825	**11.075**	**0.000**	0.593	2.895	**0.000**	**0.155**	**0.000**	**0.054**	****
FTIC/Transfer student		3.238	1.066	1.488	**10.741**	2.376	0.329	0.460	3.318	0.734	1.397	**10.079**	2.229	**7.216**	1.596	**0.221**
Good academic standing		**68.497**	**0.180**	3.642	1.816	**0.267**	**0.003**	**0.053**	**0.027**	**0.004**	**20.192**	**10.067**	1.480	0.499	**0.073**	**0.147**
Senior level		1.075	2.082	1.579	2.339	1.687	1.936	1.468	2.175	1.569	0.758	1.124	0.810	1.482	1.069	0.721
Excess hours		**0.013**	0.220	**0.182**	**0.024**	**0.078**	**16.496**	**13.608**	1.768	**5.803**	0.825	**0.107**	0.352	**0.130**	0.426	3.282
Full-time		1.847	**7.645**	1.431	**6.701**	**7.305**	4.140	0.775	3.629	3.956	**0.187**	0.876	0.956	4.684	**5.106**	1.090
Academic decline		0.209	****	0.662	2.234	**15.813**	****	3.169	**10.687**	**75.647**	**0.000**	**0.000**	**0.000**	3.373	**23.874**	**7.780**
Pell eligible	Yes	0.829	**33.947**	**21.195**	**9.838**	**0.263**	**40.939**	**25.561**	**11.865**	0.317	0.624	**0.290**	**0.008**	0.464	**0.012**	**0.027**
	Unknown	**0.054**	**0.085**	**0.052**	**0.024**	**0.154**	1.593	0.976	0.457	2.869	0.613	**0.287**	1.802	0.468	2.941	**6.279**
Institutional debt		0.238	2.307	1.497	1.225	0.848	**9.710**	**6.302**	**5.157**	3.570	0.649	0.531	0.368	0.818	0.567	0.692
Loans borrowed		0.304	**25.033**	**41.973**	**33.957**	0.340	**82.462**	**138.265**	**111.860**	1.120	1.677	1.357	**0.014**	0.809	**0.008**	**0.010**

Odds ratios > 5 or < 0.2 are bolded to signify a high degree of class separation (Nylund-Gibson and Choi, 2018).

The ways in which the variables combined led to the distinction of several terms used to name the classes. The combination of good academic standing, no excess hours, and part-time enrollment status formed the term *Distracted*; these variables indicate a student has slowed their progress toward degree completion and may have competing obligations. When Pell eligibility and loan borrowing were both present, the term of *High Financial Need* was applied to the latent class. Conversely, *Self-Pay* indicates the absence of Pell eligibility information (high item response probability for *unknown*) and no history of loan borrowing; when both of these characteristics are present, there is no indication of financial aid usage at the institution. The combination of academic decline and poor academic standing poses a significant barrier to degree completion and is indicated by the term *Academic Distress*. The distinction between academic decline and academic distress is critical for intervention efforts and the degree to which academic struggles impact departure. A student with academic decline but in good academic standing does not need to adhere to strict guidelines if they chose to re-enroll. Conversely, a student in academic distress will return to the university on probation and needs to raise and maintain their GPA to 2.0 to be eligible to remain at the institution. Furthermore, financial aid is inaccessible to students who are in poor standing. See Table 5 for these overarching labels, class names, and item endorsements. The latent classes are numbered and listed in order of severity of barriers for reentry.

There are similarities/overlap among several of the latent classes, in which the financial variables provided the defining distinctions. Specifically, two pairs of variables were very similar in most characteristics but differentiated by the financial variables and are discussed first. Classes One and Two were both characterized as *Distracted* but differed in *Self-Pay* and *High Financial Need*. Specifically, Class One (13.39% of the sample) was comprised of transfer students in good academic standing, not in excess hours, with part-time enrollment status, not in academic decline, no institutional or loan debt, and unknown Pell eligibility. Class One was labeled as *Distracted Self-Pay*. Class Two was the second largest class at 19.66% and shared similarities with Class One, but was non-traditional in age with a history of Pell eligibility and loan borrowing. Class Two was labeled as *Distracted with High Financial Need*. *Academic Distress* was present in both Classes Five and Six, but the two classes also differed in *Self-Pay* and *High Financial Need* as well. Class Five was a small group representing 7.97% of the sample and was characterized by traditional-aged transfer students, in poor academic standing and academic decline, not in excess hours, with no institutional debt or loan borrowing, and unknown Pell eligibility, leading to the label of *Academically Distressed Self-Pay*. Class Six (18.64%) was characterized by non-traditional-aged transfer students, in poor academic standing (moderately) at senior level, in academic decline, previous Pell eligibility, and a history of loan borrowing, receiving the label of *Academically Distressed with High Financial Need*.

The two remaining classes share some characteristics with the other classes but remain distinctively different. Class Three was the largest latent class at 32.55% of the sample and the only class not dominated by transfer students. Class Three consisted of traditionally-aged students in good academic standing, not in excess hours, in academic decline, with previous Pell eligibility and loan borrowing; this class was labeled as *Academic Decline with High Financial Need*. Class Four prevalence was the smallest at 7.25% and was characterized by non-traditional aged transfer students, in good academic standing, with excess hours, part-time enrollment status, unknown Pell eligibility, no institutional debt, and no history of loan borrowing. The characteristics of Class Four indicated students with data similar to that of a student who has attended multiple institutions and struggled to make progress on degree completion (McCormick, 2003; Johnson and Muse, 2012), and therefore was labeled as *Swirlers*.

## 4 Discussion

The goals of the study were to determine if there were latent classes within a sample of stopped-out seniors, their size and characteristics, and demonstrate how institutions can use the results of this statistical technique to inform intervention planning. We believe the goals of the study were accomplished. The results of a six-class solution best fit the stopped-out senior sample. The classes were characteristically different from each other and the results can inform intervention strategies and resource allocations.

### 4.1 Latent classes within stopped-out seniors

The literature suggested that students stop out from post-secondary education for a variety of academic, financial, and personal reasons. Previous research examined how these factors form barriers to re-entry; however, they examined and discussed the different reasons as separate phenomena (Donhardt, 2012; Lane et al., 2012; Hagelskamp et al., 2013; Schulte, 2015; Steele and Erisman, 2016; Mabel and Britton, 2017). Several studies examined the impact of financial variables, academic variables, and life circumstances on a student’s intent and ability to re-enter education, but none examined the combinations of these variables within a group of stopped-out students. This study aimed to determine the combination of factors that contributed to a student’s departure that may hinder them from returning to their education. With the available data, this latent class analysis classified a group of stopped-out seniors into six separate groups based on how they responded to academic, financial, and demographic variables combined. LCA was instrumental in uncovering the unique combinations of variables that go undetected with variable-centered statistical methods.

Consistent with previous literature, academic and financial variables were key to defining the latent classes. Additionally, transfer students dominated the stopped-out seniors and transfer status was a significant characteristic for five of the six latent classes (i.e., all classes except for Class Three), demonstrating the negative impact of mobility on degree completion as shown in previous literature (Hossler et al., 2012; Simone, 2014; Schulte, 2015; US GAO, 2017; Giani, 2019). Also, non-traditional students characterized three of the five classes for which age was a defining factor (i.e., all classes except for Class One). This is consistent with studies documenting the difficulty older students face in balancing school, life, and work demands (Lane et al., 2012; Hagelskamp et al., 2013; Steele and Erisman, 2016). The burden of the cost of education was evident within the latent classes. Three classes (i.e., Classes Two, Three, and Six) were characterized by previous need/use of financial aid, a fourth (i.e., Class Four) was defined by excess hours which increase a student’s tuition costs, and a fifth group (i.e., Class Five) will be ineligible for financial aid based on their academic standing should they return to their education. The structure and characteristics of the latent classes mirror the changing student archetypes and pathways, as well as their need for financial assistance to cover high educational costs.

Although a four-class solution was expected, the final six-class solution was an extension of the hypothesized classes. Class Five, *Academic Distress, Self Pay,* and Class Four, *Swirlers*, represented the hypothesized class composed of students with primarily academic barriers. Class Two, *Distracted with High Financial Need,* represented the hypothesized class of students with primarily financial barriers. Both Class Six, *Academic Distress with High Financial Need,* and Class Three, *Academic Decline with High Financial Need,* were classes composed of students with a combination of academic and financial barriers. The combination of financial need and academic difficulties is particularly problematic for students, because academic decline jeopardizes financial aid eligibility, and consequently the costs of education become greater. The high prevalence of students (70.85%) within classes defined as high financial need (i.e., Classes Two, Three, and Six), underscores the literature surrounding the heavy reliance on financial aid in the current landscape of higher education (College Board, 2018; Radwin et al., 2018; Snyder et al., 2019). Lastly, the *Distracted, Self-Pay* (Class One) group was similar to the hypothesized class of students with no significant barriers academic or financial barriers.

One main limitation to this study was the need to work within the data structures readily available to institutions, but results of this LCA are encouraging. Only a small percentage (13.93%, Class One) of the sample fell into the category of students with no significant barriers identified within institutional data. This indicates that the majority of barriers experienced by students are detectible to institutions within the data that is on hand and meaningful analysis can occur within these limitations. The application of LCA allowed for the identification of six distinct groups of students with varying levels of barriers that require different tactics for re-enrollment efforts.

### 4.2 Intervention strategies guided by latent class analysis

Although some of the latent classes are small and others overlap in several characteristics, the distinctions between the six classes can guide an institution toward decisions on the allocation of resources and intervention strategies. For instance, an institution may want to begin intervention efforts with the students facing the least number of barriers, which in turn would require fewer resources. In this case, the *Distracted Self-Pay* (Class 1) and *Distracted with Financial Need* (Class 2) may be the groups the institution chooses to target first for re-enrollment. These two classes faced no significant academic barriers; their departure could have been due to life circumstances or financial need, and they may be the easiest to re-enroll with financial assistance or incentives. Alternatively, students in academic distress (*Academic Distress with High Financial Need*—Class 6 and *Academic Distress Self-Pay*—Class 5) face larger barriers to re-entry, especially those in financial need. Poor academic standing and academic decline not only hinder progress toward degree completion related to coursework, but these students are also not able to qualify for financial aid. Students in academic distress require many resources from the institution and may be more difficult to rehabilitate. The two remaining classes, *Swirlers* (Class 4) and *Academic Decline with High Financial Need* (Class 3), will require additional resources to re-enroll than the distracted students, but less than the academically distressed students; the institution can consider these classes for a second-level intervention plan.

Table 7 outlines the possible intervention strategies for each latent class and how the institution can use the characteristics of each class to determine priorities. The interventions strategies included are not exhaustive, but cover academic, financial, lifestyle/social support, and resource allocation. Although several of the intervention strategies are recommended for re-engaging all of the stopped-out seniors, others are recommended specifically related to the characteristics of the latent classes. Here we highlight some important strategies. First, global strategies would include a degree audit (to assess the student’s earned credits and what remains in their degree plan), individualized communication from their academic department (to encourage student connection to the campus), financial aid counseling, waving the reapplication fee, a returner/completer scholarship, and mentoring programs (to deepen student connection to the campus community). Second, other intervention strategies such as extend hours of operation for key areas and connection to lifestyle services would be more focused on reaching out to students within the latent classes dominated by non-traditional students. Third, within the largest Class 3, *Academic Decline with High Financial Need*, and the *Academic Distress* classes that would require the most resources (i.e., Classes 5 & 6), the institution can target efforts on re-enrolling students in high demand degrees. The remaining strategies relate to the characteristics of the specific latent classes and their details can be found in Table 7.

**Table 7:** Possible Intervention Strategies for Each Latent Class.

	Class 1: distracted, self-Pay	Class 2: distracted, high financial need	Class 3: academic decline, high financial need	Class 4: *Swirlers*	Class 5: academic distress, self-pay	Class 6: academic distress, high financial need
**Academic**						
Degree audit: assess catalog requirements, what courses remain	X	X	X	X	X	X
Academic plan change: explore additional degree plans based on student’s completed coursework	X	X		X		
Modality and location options: promote online/hybrid offerings and course availability at branch campuses	X	X		X		X
Tutoring services: connect students to university, college, and department tutoring services			X		X	X
Individualized communication from academic department: connect a student with a faculty member from their chosen academic department	X	X	X	X	X	X
**Financial**						
Waive reapplication fee: do not require returning students to pay an application fee upon reapplying to the university	X	X	X	X	X	X
Financial aid counseling: assistance with completing a FAFSA, examine eligibility based on prior enrollments and academic status, discuss payment plans	X	X	X	X	X	X
Institutional debt forgiveness: offer debt forgiveness on prior balances, up to a specific amount		X				X
Returner/Completer scholarship: offer a scholarship to students who return to complete their degree	X	X	X	X	X	X
**Lifestyle/Social support**						
Extend hours of operation for key areas: offer extended hours for the returning students to complete admissions requirements, financial aid activities, academic counseling, and other key tasks		X		X		X
Connect to lifestyle services: connect returning students to campus resources such as childcare services, financial and money management services, transfer center, multicultural center, first-generation resources, and non-traditional student services		X		X		X
Mentoring programs: pair returning students with peer mentors or professional mentors	X	X	X	X	X	X
**Resource allocation**						
Target high demand degrees: focus on reenrolling students in degrees that align with high demand jobs, emphasize career opportunities with completion			X		X	X

The Bivariate Residual (BVR) analysis results on the 6-class model indicates that this model may not entirely encapsulate the complexity of interactions between certain variables, and more latent classes can be considered. However, practical challenges arise when distinguishing between classes and implementing interventions beyond 6 classes. While there was some support for a 7-class or 8-class model through the continued decreasing of AIC, BIC, and SBIC, the additional latent classes became difficult to substantively distinguish from already existing classes. Additionally, the interventions matching the barriers faced by the additional latent classes were the same as those proposed for classes within the 6-class model. The number of latent classes is determined by utilizing a combination of both statistical and substantive model checking routines (Muthén, 2003). Balancing model fit and real-world applicability is crucial. As a result, the 6-class model was chosen based on a combination of fit indices and practical considerations.

### 4.3 Limitations and future research

The limitations of this study centered on the variables used in the latent class analysis. Many of the key data points contributing to education departures identified in the literature, work, family, health (Donhardt, 2012; Lane et al., 2012; Hagelskamp et al., 2013; Schulte, 2015; Steele and Erisman, 2016; Mabel and Britton, 2017) were not available to this institution and therefore not included in this study. Other institutions may have a broader range of information on their students and are able to incorporate that data into their analyses. Although one of the aims of this study was to demonstrate how to work within these limitations, including data related to all relevant aspects of the research question is ideal. The inclusion of additional variables will increase the ability of the latent classes to reflect the full range of issues contributing to attrition and further inform the institution on possible intervention efforts. Institutions cannot mitigate all reasons for departure; the inclusion of non-malleable variables in the analysis can further help an institution sort out students they want to allocate resources toward and those outside their scope of influence. Future research should include these additional variables when available and institutions should consider collecting additional information on all of their students so that analyses and conclusions reflect the full breath of the student’s experience and challenges. Future research is needed confirm similar latent classes from data at other institutions.

Future research can apply an alternative approach to this study, by examining the latent classes that exists within all seniors. Rather than examining the latent classes that exist among only stopped out seniors, researchers can instead use the entire population of seniors and then determine whether or not students tend to stop-out at different rates across the classes. This method can aid in preventative measures to stop student attrition. Given the number of stopped-out seniors that arrived at the university as transfer students, this method may allow for significant preventative measures for those students. The goal of this study was to mitigate the impact of attrition after it has occurred, but the findings and approach can be applied to preemptive intervention planning.

An additional limitation to this study is the inherent nested or multi-level structure of academic data (Fagginger Auer et al., 2016). Traditional latent class analysis has an assumption that the observations are independent of one another, but this is not true for data with a nested structure (Henry and Muthén, 2010). Ignoring the nested or multilevel nature of a data set has shown to bias parameter estimates and standard error estimations and increase Type I error rates (McCoach and Adelson, 2010). Within this study, students are nested within colleges, departments, and majors. Multilevel latent class analysis (MLCA) accounts for the nested structure of academic data and allows for the examination of how level-2 units (e.g., colleges, or departments) affect latent class membership at level-1 (i.e., students) (Henry and Muthén, 2010; Cabrera et al., 2014; Allison et al., 2016). This sample of stopped-out seniors included students from 11 colleges, 76 departments, and 143 majors. Some colleges and majors were overrepresented within the stopped-out senior population, indicating the potential influence of this nested structure on stopping out and therefore the latent classes. The clusters within the sample are unbalanced; the college clusters range in size from 57 to 1,030 students, the department cluster sizes range from 1 to 345, and the academic plan clusters range from 1 to 297 students. Zhang et al. (2014) found that cluster sizes for MLCA should be at least 40 in size and that complex models need even larger cluster sizes to ensure accurate class enumeration. Nevertheless, future research should explore the impact of the nested structure on student-level latent classes through MLCA.

### 4.4 Conclusion

The purpose of this study was to use latent class analysis to uncover the underlying structure within a group of stopped-out students and demonstrate how this statistical technique enhances intervention planning. The six latent classes of stopped-out seniors formed mainly around the financial and academic variables, and the characteristics of the classes align with previous research on students that have stopped out. Pell eligibility and loan borrowing were instrumental in forming the latent classes, both in their presence (high financial need) and absence (self-pay). This indicated that a majority of the stopped-out seniors in this study experienced the impact of the cost of education and are subject to the negative consequences of loan debt without the completion of a degree (Gladieux and Perna, 2005; Tierney, 2012; U.S. Department of Education, 2015; Itzkowitz, 2018). Besides, transfer students dominated five of the six latent classes, underscoring how the complex pathways students take to degree completion are contributing to their departure (Peter and Forrest Cataldi, 2005; Hossler et al., 2012; Simone, 2014; US GAO, 2017; Giani, 2019). Additionally, severity of academic challenges also was key to separating the classes. Overall, the identification of subgroups within a larger sample of stopped-out seniors allows institutions to identify specific intervention strategies and make decisions on where to focus efforts and resources. The variables included in this study are not unique to this institution, and the sample reflected key issues identified in the literature, therefore other public institutions would benefit from a similar approach within their stopped-out student populations.

The results and methods of this study have implications for institutions, higher educational professionals, and institutional researchers. The application of a person-centered statistical approach, latent class analysis, allowed for the discovery of how various academic, financial, and demographic variables combined within stopped-out seniors; and the resulting six-class solution is an advantage over variable-centered approaches. Furthermore, the ability to partition a large and daunting number of stopped-out students into meaningful subgroups is critical and informative to the formation of strategies to re-engage the students and make informed decisions around resource allocations. The ability to work within the limited data landscape of what is readily available to institutional researchers highlights a way to move forward when so much is unknown about students. Last but not the least, the segmentation of students into characteristically different groups is critical to the application of tailored intervention strategies that increase efficiency and effectiveness, without increasing the amount of resources needed.

## Data availability statement

The data analyzed in this study is subject to the following licenses/restrictions: the data for analysis is institutional data at the student level without personal identifiers; however, it was derived from a bigger dataset that contains sensitive and protected content that cannot be released to the public per FERPA. Requests to access these datasets should be directed to cassie.clough@gmail.com.

## Ethics statement

The studies involving humans were approved by the Institutional Review Board (IRB) at University of North Texas (UNT). The studies were conducted in accordance with the local legislation and institutional requirements. Written informed consent for participation was not required from the participants or the participants’ legal guardians/next of kin in accordance with the national legislation and institutional requirements.

## Author contributions

CW: Conceptualization, Investigation, Methodology, Writing – original draft, Writing – review & editing, Data curation, Formal analysis, Project administration, Resources, Visualization. QC: Conceptualization, Investigation, Methodology, Supervision, Writing – original draft, Writing – review & editing. NB: Writing – review & editing.
